# Navigating the labyrinth of healthcare workers' mental health

**DOI:** 10.3389/fpubh.2025.1699755

**Published:** 2025-11-14

**Authors:** Hai-long Zhang, Qin-yuan Zhang

**Affiliations:** 1 Shuyang Hospital of Traditional Chinese Medicine, Shuyang, Jiangsu, China; 2 Shanghai Key Laboratory of Psychotic Disorders, Shanghai Mental Health Center, Shanghai Jiaotong University School of Medicine, Shanghai, China

**Keywords:** healthcare workers, mental health, stress-coping-accumulation-imbalance, anxiety, depression

## Abstract

The prevalence of mental health issues such as burnout, anxiety, and depression, and the negative impact on both healthcare workers and the healthcare system are notably high. Here, we introduced a dynamic model of mental health that includes stress, coping mechanisms, and resilience, and incorporates individual, organizational, and socio-cultural factors. It emphasized the importance of addressing moral dilemmas, high workloads, and systemic deficiencies that contributed to mental health risks. Additionally, innovative tools like AI-based predictive models were used to identify at-risk individuals early. In a word, special attention was called for comprehensive support systems, national policies, and cross-cultural research to improve healthcare workers' mental wellbeing. Ultimately, we stressed the need for systemic reforms and individualized interventions to safeguard the mental health of healthcare workers and ensure better patient care. This article highlighted the global mental health challenges faced by healthcare workers, especially in the context of the COVID-19 pandemic and other health crises.

## Introduction

The mental health of healthcare workers (HCWs) has been a growing concern nowadays, exacerbated by the ongoing challenges of the COVID-19 pandemic and other health crises (1, 2). Notably, the COVID-19 pandemic highlights the critical role of HCWs while simultaneously amplifying their psychological burden (3). HCWs, including doctors, nurses, and other allied health staff, have consistently reported high levels of stress, burnout, anxiety, and depression. Studies indicate that HCWs are much more susceptible to mental health disorders than the general population. For instance, a global meta-analysis found that 23.2% of HCWs experience symptoms of anxiety, with 22.8% suffering from depression and 34.3% reporting insomnia during the pandemic's peak (4).

This crisis has profound implications both for the individuals affected and for healthcare service. Poor mental health among HCWs is associated with decreased work performance, increased medical errors, and poorer patient outcomes (5). Therefore, addressing the mental health challenges among HCWs is essential not only for their wellbeing but also for maintaining the effectiveness and sustainability of healthcare systems worldwide (6).

Facing the ongoing strain on healthcare systems, it is crucial for us to clarify the complex factors leading to mental health issues and implement targeted interventions and policy changes to support the mental health of HCWs.

## Model of a dynamic “stress-coping-accumulation-imbalance” process

**Figure 1:**
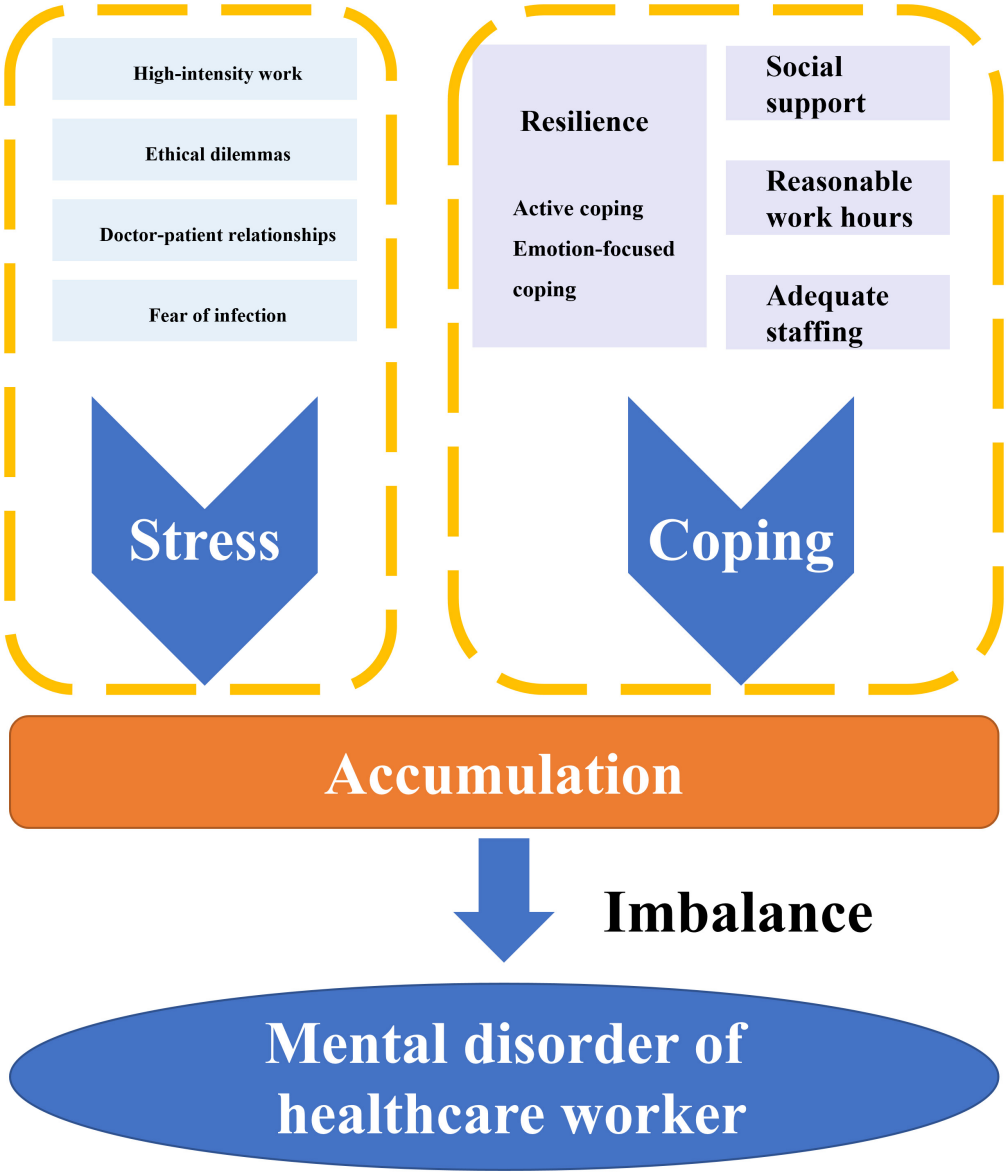
“Stress-coping-accumulation-imbalance” process.

HCWs are facing a multitude of stressors in their daily professional lives, from the emotional toll of patient care to the physical and organizational demands of their own roles. To understand the mental health challenges they encounter, it is essential to view these stressors as part of a dynamic, ongoing process but not as isolated events. This process can be described through a “stress-coping-accumulation-imbalance” framework, which integrates multiple levels of influence—individual, occupational, organizational, and socio-cultural—on HCWs' mental health (Figure 1).

At the core of this model are stressors, which can be broadly categorized into workload-related, ethical, and interpersonal challenges. First and foremost, high-intensity work is of vital importance. HCWs often experience high-intensity work involving long hours, frequent night shifts, and insufficient staff, which creates chronic stress (7). This stress is compounded by ethical dilemmas, such as decisions about resource allocation or end-of-life care, which frequently place workers in morally distressing situations (8). Furthermore, conflicts in doctor-patient relationships and exposure to patient suffering exacerbate stress levels, leading to emotional exhaustion (9). Besides, fear of infection and isolation measures during the pandemic may present more significant hazards and risks on HCWs (10). These stressors not only impact individual wellbeing but also ripple through healthcare systems, contributing to broader organizational challenges, such as resource shortages and lack of support (11).

In the model, the individual's coping mechanisms and resilience play a vital role in how these stressors are managed. Coping strategies can vary, with some individuals employing active coping—such as seeking social support or using problem-solving techniques—while others may resort to avoidance or emotion-focused coping strategies that may lead to maladaptive outcomes (12). The concept of resilience is crucial in this context, as it determines how well individuals can bounce back from adversity. However, resilience is not static. It is influenced by personal characteristics, social support, and workplace environment (13). Also, social support from colleagues, supervisors, and family members can serve as a significant protective factor, which can mitigate the negative impacts of work-related stress (14).

However, these stressors can deplete the individual's resilience reservoir as they accumulate over time. If coping resources are insufficient to replenish this reservoir, imbalance occurs, leading to mental health problems such as burnout, depression, and anxiety. This accumulation effect underscores the need for timely interventions to restore balance and protect mental health (15). Thus, the “stress-coping-accumulation-imbalance” process highlights the dynamic and cumulative nature of HCWs' mental health challenges, emphasizing the importance of both individual resilience and organizational support in mitigating mental health risks.

## Precise identification and prediction of mental health risks in HCWs

Identifying and predicting mental health risks in HCWs is crucial for timely intervention and preventing long-term psychological damage. Given the complex, dynamic nature of stressors and coping mechanisms, precision in detecting at-risk individuals can enable healthcare systems to provide targeted support and reduce the impact of mental health problems. Advances in data science and psychology have paved the way for more effective identification tools, but challenges remain in refining these methods to improve accuracy and applicability across diverse healthcare settings.

As for screening tools and early identification, standardized mental health screening tools such as the Patient Health Questionnaire (PHQ-9) are widely used to detect symptoms of depression, anxiety, and burnout in HCWs (16). These tools have demonstrated effectiveness in identifying individuals who are experiencing psychological distress, but they may not fully capture the complexity of HCWs' emotional and cognitive experiences. For instance, while these questionnaires are useful for detecting major depressive symptoms, they often fail to account for subtle forms of psychological distress, such as moral injury or compassion fatigue, which are unique to healthcare professionals (17).

When it comes to predictive models and artificial intelligence, recent advancements in predictive analytics, including machine learning and artificial intelligence (AI), offer promising new approaches for identifying mental health risks in HCWs. By analyzing large-scale datasets—ranging from electronic health records to self-reported surveys—AI models can uncover patterns and predict the likelihood of mental health issues before they manifest fully (18). Of course, the predictive AI model is expected to exclusively analyze work-related healthcare records rather than HCWs' personal health records (PHRs). Besides, the AI tool should be designed as a voluntary, opt-in prevention resource rather than a mandatory system. HCWs can autonomously choose to participate, or withdraw at any time without impacting their employment status or professional evaluations. Finally, multi-layered privacy safeguards are needed, including data de-identification, technical safeguards, independent institutional oversight and so on.

Current research has begun exploring the application of AI and the Internet of Things (IoT) in mental health monitoring (19). “IoT” specifically denotes a set of research-specific, purpose-built connected devices deployed to collect objective, non-intrusive data that complements self-reported mental health assessments for HCWs. Critical to this design is that all IoT data collection is conducted with consent from participating HCWs and they retain full control. For instance, the EmoPulse system enables real-time psychological diagnosis by integrating facial expression analysis with psychometric assessments (20).

However, these systems exhibit limitations in multimodal data integration and personalized risk prediction. Key innovations focus on multimodal biopsychological data integration and analysis, constructing comprehensive digital mental health profiles for HCWs through synthesized physiological data (e.g., heart rate variability, sleep patterns, electrodermal activity) from wearables, clinical records from electronic health systems, voice/text-derived emotional and cognitive patterns via natural language processing, and facial expression recognition (21, 22). Deep learning architectures including Recurrent Neural Networks and Transformer models fuse these heterogeneous data streams to identify early biomarkers and behavioral patterns associated with psychological stress, burnout, anxiety, and depression.

While the potential for precise identification and prediction is significant, several challenges remain. The diversity of healthcare settings—ranging from hospitals to primary care practices—necessitates adaptable models that can accommodate different work environments and stressor profiles. Furthermore, the ethical considerations surrounding data collection, particularly regarding sensitive mental health information, require careful attention to ensure confidentiality and informed consent (23). The core objective of this work is to support HCWs and, through improved HCW wellbeing, ultimately enhance patient care; critically, it is not intended for, nor should it be used by, hospital administration as a tool for exploitative manner. Moving forward, enhancing the granularity and specificity of predictive models will be key to better understanding and addressing the mental health risks faced by HCWs.

## Discussion

The mental health of HCWs is a critical global issue that demands urgent attention. As frontline responders to public health crises, HCWs are exposed to chronic stressors that can lead to significant psychological distress, including burnout, anxiety, and depression. The “stress-coping-accumulation-imbalance” model provides a comprehensive framework for understanding these challenges, highlighting the dynamic interplay between individual vulnerabilities, occupational stressors, and organizational factors.

The development of accurate, AI-driven identification and prediction tools offers a promising path forward in safeguarding the mental health of HCWs. By improving the precision of risk assessments, healthcare systems can intervene earlier, mitigate the harmful effects of stress, and ensure that HCWs receive the support they need to thrive. Notably, the use of biomarkers and predictive AI is always intended to complement—not replace—HCWs' subjective self-assessment of burnout. These tools should be used as complementary tools rather than substitutive ones to HCWs' subjective self-assessment of wellbeing—their self-judgment remains the final authoritative standard throughout. The rationale for integrating such supplementary tools lies not in doubting HCWs' perceptions, but in addressing well-documented barriers to timely burnout disclosure in frontline settings: HCWs often normalize chronic fatigue and distress as “role-inherent” due to professional culture, leading to unrecognized early symptoms. Compounding this, burnout's gradual onset means pre-subjective physiological and behavioral signals emerge weeks before conscious awareness, and biomarkers/AI models act as non-intrusive early warning systems to capture these signals, reducing reporting bias and missed cases without undermining self-report's clinical value. To preserve HCW autonomy, all data should be accessible only to HCWs and their support providers (not administrators) to prevent misuse.

Addressing the mental health of HCWs requires continued research to refine our understanding of the complex factors that contribute to psychological distress and to develop effective, scalable interventions. As the global healthcare workforce faces increasing challenges, future research should focus on innovative solutions that integrate psychological, organizational, and systemic perspectives, while ensuring that findings are applicable across diverse healthcare settings.

One key area for future research is the exploration of longitudinal studies that track HCWs over time, assessing how stressors accumulate and how resilience evolves in response to different interventions. Such studies could provide insights into the temporal dynamics of mental health issues, identifying critical points for intervention before burnout, anxiety, or depression becomes entrenched (24). Moreover, cross-cultural studies are essential to understand how diverse healthcare systems and cultural norms influence the mental health of workers. Comparative research across countries with varying levels of resource allocation, organizational support, and social security systems could yield valuable information on effective strategies and policies for reducing stress (25).

While current interventions offer some relief, there is a pressing need for more effective, targeted strategies, particularly in the form of systemic changes and proactive support systems. Future research should focus on longitudinal studies, cross-cultural comparisons, and the development of AI-driven tools to predict and mitigate mental health risks. Ultimately, policy initiatives must prioritize HCWs' mental health to safeguard both their wellbeing and the quality of care provided to patients.

## Data Availability

The original contributions presented in the study are included in the article/supplementary material, further inquiries can be directed to the corresponding author.
